# Kienböck’s disease in an 11-year-old girl: a case report

**DOI:** 10.11604/pamj.2019.34.95.19116

**Published:** 2019-10-16

**Authors:** Ahmed Amine Mohseni, Mohamed Zairi, Rim Boussetta, Walid Saied, Sami Bouchoucha, Nebil Nessib

**Affiliations:** 1 Department of Orthopaedic Surgery, Children’s Hospital Pediatric Orthopedic Surgery Department Béchir Hamza, Tunis, Tunisia

**Keywords:** Pediatric, lunatum, kienböck

## Abstract

Kienböck disease is a pathology that remains uncommon in children, therefore the number of published cases of Kienböck’s disease before skeletal maturity is limited. The etiology of Kienböck’s disease is still controversial. Although many therapeutic methods are described in the literature. There is no consensus treatment for this pathology. We describe a case of Kienböck’s disease of an 11-year-old girl who presented with avascular necrosis of the lunate bone confirmed by the radiologic pattern. And who was treated with 10 weeks of splinting with satisfying clinical outcome. Wrist pain and other symptoms resolved after 2 months. A magnetic resonance imaging (MRI) confirmed partial revascularization of the lunate. After 18 months of follow-up the patient remains asymptomatic.

## Introduction

Kienböck's disease is an ischemic necrosis of the lunate which usually occurs on young adults between 20 and 40 years old, but it rarely affect teenagers and children. The term of infantile lunatomalacia is used when the disease affects children under the age of 13 while juvenile lunatomalacia defines the patients above 13 years old to the end of skeletal maturity [1]. There is only a small number of published cases of Keinböck disease before skeletal maturity. Nineteen cases have been reported in the literature [2-4]. We describe a case of an 11-years-old girl injured left (non-dominant) wrist, in a minor trauma who was treated with 10 weeks of splinting.

## Patient and observation

**Figure 1:**
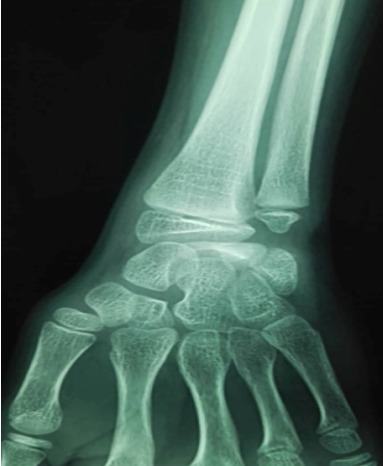
Face radiograph shows collapse of the lunate

**Figure 2:**
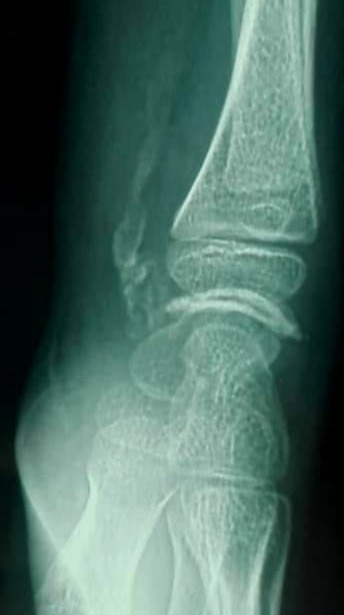
Profil radiograph shows collapse of the lunate

**Figure 3:**
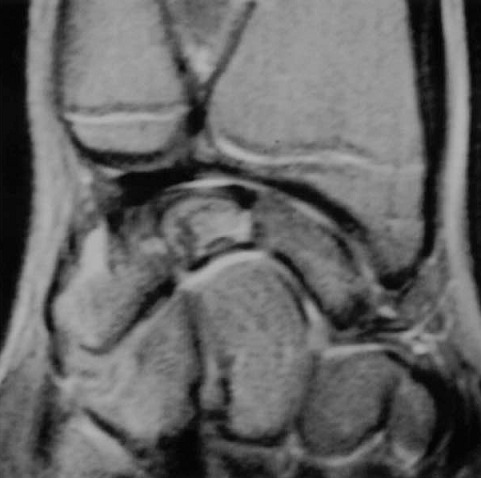
Magnetic resonance imaging (MRI) shows beginning of revascularization of the lunate

**Figure 4:**
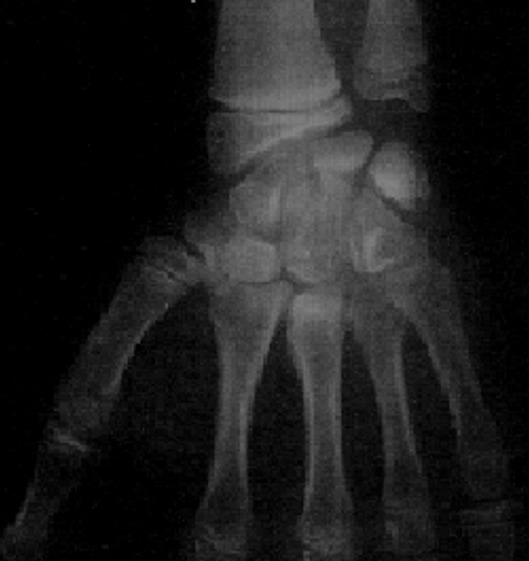
Face radiograph shows healing of the lunate

**Figure 5:**
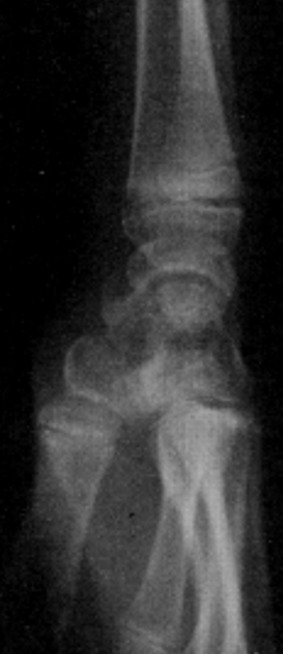
Profil radiograph shows healing of the lunate

An 11-year-old girl who injured her left (non-dominant) wrist when she fell from her own height. The patient has no previous history of fracture or dislocation or any other medical pathology. Tenderness on left wrist was detected on physical examination. Neurologic and vascular examinations were normal. Tightening force compared to the other side significantly decreased and a limitation in wrist movement with 60° extension and 55° flexion but pinch strength test was normal. The X-rays showed sclerosis and total collapse of the lunate (Figure 1, Figure 2) posing Kienböck’s disease which was graded according to Lichtman’s classification as stage IIIA. The carpal height ratio of the affected wrist was 0.43 lower than unaffected wrist 0.54, a normal value being 0.54-0.03 [5]. A conservative treatment was therefore recommanded with splinting during 10 weeks and rest (sports of any kind were not allowed) during 5 months. After 2 months, wrist pain and other symptoms were resolved and she has regained full wrist mobility. Three months later an MRI showed a remodeling of the lunate (Figure 3) and the practice of the sport (simple exercises at school) was allowed gradually. After 18 months of follow-up the patient remains asymptomatic and radiologic patterns regaining of lunate’s height (Figure 4, Figure 5).

**Statement of informed consent:** we contacted the parents of the child and we obtained the agreement to publish the case for scientific research.

## Discussion

Since the first description of Kienböck disease in 1910, it is still an uncommon pathology in children. It is a process of unknown etiology resulting in osteonecrosis of the lunate bone generated by a combination of anatomical factors (ulnar variance negative) and traumatic factors (chronic overstress of the bone) [1, 2] which create a hyperpressure in the lunate bone. An interruption of arterial vascularization, subcortical microfractures, or decreased intraosseous venous return [1, 2] explain some cases affecting children practicing sport that overstress the hand such us gymnastics [1, 2] and baseball. In our case the fortuitous discovery of the KienbÖck disease was not related to any particular sport’s activity.

In the literature only 19 cases have been reported, most of them are described in case report [1, 3]. There are only three series [3, 4] that describe respectively 4, 5 and 2 cases. The treatment of Kienböck’s disease has been extensively debated and there is still no consensus. Most of the reported observations in the literature suggest that revascularization of the lunate can be obtained in children by conservative treatment (rest and prolonged immobilization of the wrist) even in advanced cases [3-6]. This has been confirmed in our case wrist pain and mobility were resolved after 2 months. The improvement of clinical signs allows us to continue conservative treatment despite the advanced stage of the disease (classification IIIA of Lichtman).

De Smet [6] described also a stage IIIA Lichtman case successfully treated by immobilization. Herzberg [1] and Mathieu [2] reported that MRI and computerized tomography (CT) are interesting in treatment’s monitoring which shows a slight remodeling of the lunate and prevents extra immobilization that deprives the child of normal range of motion and sports activities. In our case the beginning of revascularization of the lunate proved by the MRI around 5 months after the beginning of the treatment allows to return to normal activity (Figure 3).

We observed complete healing of the lunate in standard x-rays (Figure 4, Figure 5) after 18 months of follow-up. Many authors [1-3, 6] agree that surgical treatment is indicated in the failure of conservative treatment. Several techniques have been described and the goal is to unload the lunate and to decrease the compressive forces: radial shortening the most often described [1], temporary scaphotrapezoidal joint fixation and distal radius definitive epiphysiodesis. Compared to adults, the child have better results, probably because the potential of the lunate to revascularize is greater [3]. Blachier [7] reported a case of bilateral stage III Kienböck’s disease in a 14-years-old girl who was treated with bilateral radial shortening and at the 4 months follow-up evaluation lunate was observed on radiologic patterns and the patient was pain free.

Irissarri *et al.* [4] obtained a good clinical and radiological outcome in three boys with radial shortening osteotomy stabilized with a palmar plate after a 5 years follow-up. Radial shortening biomechanically unload the lunate by shifting the load toward the ulna and the good clinical outcomes can be explained by the reduction of the actual stress across the distal articular surface of the radius. Jorge-Mora *et al.* [5] reports a new surgical technique based on epiphysiodesis of the distal radius with the subsequent diminution of compressive forces acting in the lunate. Yasuda *et al.* [8] 1998 Shigematsu [9] 2001 treated Kienböck's disease in 11 and 12 years old girls with temporary scaphotrapezoidal joint fixation. Clinical and radiological outcome was satisfying in both cases, 20 and 14 months respectively after surgery.

## Conclusion

We agree with most authors to recommend conservative treatment of Kienböck's disease in children as a first approach. The satisfying clinical results allow to continue the conservative treatment and the beginning of the lunate’s revascularization concluded by the MRI authorized the return to normal activities. The surgical treatment should be considered if there is a mention of persistence pain and/or wrist stiffness.

## Competing interests

The authors declare no competing interests.
